# Place-Based Structural Disadvantage, Lethal Police Violence, and Adverse Birth Outcomes Among Black Birthing People

**DOI:** 10.1007/s11524-026-01131-w

**Published:** 2026-08-17

**Authors:** Emma A. Blackson, Briana M. Williams, Jé Judson, Caryn Bell, Maeve E. Wallace

**Affiliations:** 1 Department of Social, Behavioral, and Population Sciences, School of Public Health & Tropical Medicine, Celia Scott Weatherhead, Tulane University, New Orleans, LA USA; 2 Program for Research On Men’s Health, Department of Family and Community Health, Program for Research On Men’s Health Office 220L, University of Pennsylvania School of Nursing, Claire M. Fagin Hall, 418 Curie Boulevard, Philadelphia, PA 19104-4217 USA; 3 Department of International Health and Sustainable Development, Celia Scott Weatherhead School of Public Health & Tropical Medicine, Tulane University, New Orleans, LA USA; 4 Resound Research for Reproductive Health, Austin, TX USA; 5 Division of Epidemiology and Community Health, University of Minnesota, Minneapolis, MN USA; 6 Department of Health Promotion Sciences, Mel and Enid Zuckerman College of Public Health, University of Arizona, Tucson, AZ USA

**Keywords:** Lethal police violence, Structural disadvantage, Structural racism, Black maternal health, Preterm birth, Low birth weight

## Abstract

Structural racism and lethal police violence shape community environments, particularly in urban and urban-adjacent contexts, and may influence reproductive health. While disparities in birth outcomes among Black populations are well documented, fewer studies have examined how these structural and state-linked exposures jointly pattern risk for adverse birth outcomes. Using 2020 National Center for Health Statistics natality data and Mapping Police Violence data (2015–2019), we analyzed associations between county-level lethal police violence, a multidomain. Structural Racism Exposure Index (SREI), and low birth weight (LBW) and preterm birth (PTB) among non-Hispanic Black birthing people in the United States (N = 407,840). Incidence rate ratios (IRRs) were estimated using. Poisson and multilevel Poisson models, adjusting for maternal sociodemographic characteristics, with multiple sensitivity analyses assessing robustness. Higher levels of structural disadvantage were consistently associated with greater rates of LBW and PTB. Associations between lethal police violence and adverse outcomes were smaller and varied across model specifications. One interaction suggested a possible elevation in PTB in counties with both high police violence and medium structural disadvantage, though interaction findings were limited and should be interpreted cautiously. Findings provide suggestive evidence that structural disadvantage and policing are contextual factors linked with birth outcomes among Black birthing people. Results highlight the importance of examining structural and place-based conditions when assessing reproductive inequities and underscore the need for further research on how these contextual exposures shape Black reproductive well-being.

## Background

Policing in the United States is one of the clearest modern manifestations of structural racism, with origins in slave patrols and deep entanglements with racialized social control; Black individuals are disproportionately subjected to police surveillance, criminalization, and state-sanctioned violence [1–5]. These exposures are not isolated incidents, but embedded within a broader context of systemic harm that accumulates across time and generations [6–8]. While the direct loss of life is often the most visible outcome, the ripple effects of police violence extend far deeper, shaping the health and well-being of entire communities [2, 4, 9]. Importantly, lethal police violence is spatially patterned in the U.S., with concentrated exposure in urban counties where policing density and community surveillance are highest [4, 10–13].

For Black birthing people, these systemic stressors may translate into significant reproductive health consequences. Adverse birth outcomes such as low birth weight (LBW) and preterm birth (PTB) remain disproportionately high among Black individuals [14–16]. These disparities are not because of individual behaviors or biological differences, but rather the cumulative relationships of structural racism, stress, and state violence [1, 7, 17–20]. Recent research has linked exposure to different aspects of policing, such as fatal police violence, proactive policing, and police stops, with increased risks of PTB, highlighting police violence as a key social determinant of health, not just a criminal justice issue but a public health crisis [14, 16, 20–25].

Structural racism, the interlocking systems and policies that create and maintain racial inequities, further compounds these risks [6]. It operates across multiple domains such as housing, education, employment, criminal justice, transportation, and access to care, producing a cumulative disadvantage that harms health at the population level [1, 6]. Police violence and structural racism are not separate forces; instead, they interact and reinforce one another, shaping the environments in which Black families live, work, and give birth [2, 8].

Despite growing attention to the relationship between population health and policing, few studies take a Black-centered epidemiologic approach that resists comparison to White outcomes as normative [8]. Much of the literature frames racial disparities through a deficit lens, obscuring the structural conditions that drive inequity [1, 7]. This study instead centers Black birthing people as the population of interest and frames the inquiry around systems of exposure, namely, county-level police violence and structural disadvantage. County-level indicators capture policy environments, policing jurisdictions, and structural conditions that shape community health. These contextual factors are particularly relevant in urban and urban-adjacent settings, where spatially patterned structural disadvantage and police presence are concentrated.

We aim to (1) assess the association between county-level lethal police violence and low birth weight (LBW) and preterm birth (PTB); (2) assess the association between county-level structural disadvantage, measured through the Structural Racism Exposure Index (SREI), and these outcomes; and (3) estimate whether associations between lethal police violence and birth outcomes differ across levels of structural disadvantage.

We draw on national birth record data and Mapping Police Violence (MPV) database data to examine how these exposures are associated with LBW and PTB. By using the Structural Racism Exposure Index (SREI) and population-level fatal police violence rates (PVrate), this study investigates how overlapping systems of structural harm shape reproductive outcomes across place, urban public health, and policy context. Rather than seeking to merely close health gaps between racial groups, we center a Black analytic frame to illuminate how state violence threatens reproductive justice [26]. We predict that higher exposure to fatal police violence and greater structural disadvantage will each be independently and jointly associated with increased risk of low birth weight and preterm birth among non-Hispanic Black birthing people. In doing so, this paper contributes to a growing body of scholarship that positions policing and structural racism & disadvantage as critical upstream determinants of perinatal health inequity, particularly in urban and urban-adjacent contexts [4, 8, 23, 24].

## Methods

### Data Sources and Exposures

This study uses birth records from the National Center for Health Statistics (NCHS) 2020 restricted-use natality file, linked to two county-level structural exposure datasets: (1) the SREI developed by Dyer et al., and (2) fatal police violence data from the Mapping Police Violence (MPV) project.

The SREI was developed initially by Dyer et al. as a multidimensional, census-tract-level measure of structural racism. It encompasses nine domains of structural inequity, including housing, education, employment, income and poverty, criminal justice, transportation, and social cohesion [27]. Although race-neutral in construction, prior research conducted by our team demonstrated that the SREI operates more accurately as a measure of structural disadvantage, capturing community-level deprivation that is disproportionately associated with racially marginalized populations [28]. We acknowledge this limitation while centering a Black-analytic frame to capture within-group variation in structural disadvantage.

For this study, we aggregated the original tract-level SREI data to the county level by calculating population-weighted means across all census tracts within each county. This aggregation was necessary to enable linkage with the natality data, which includes geographic identifiers at the maternal county of residence. To facilitate comparative analyses, the resulting county-level SREI scores were categorized into tertiles (low, medium, high) based on national percentile distributions. Police violence data were obtained from MPV, an independent database that documents all known instances in which a law enforcement officer, on or off duty, used lethal force that resulted in the death of a civilian. This includes deaths by firearm, vehicular force, and “suicide by cop” incidents, but excludes deaths caused by vigilantes or private security not affiliated with law enforcement. MPV aggregates data primarily through media reports using automated keyword detection, manual coding, and a two-step researcher verification process [29]. While comprehensive, the dataset includes only fatal incidents and likely underestimates the full scale of police violence.

To assign county-level exposure to police violence, we used ArcGIS Online to spatially map each MPV incident to U.S. counties. This geospatial linkage was performed using the most recent 5-year (2015–2019) TIGER/Line shapefile from the U.S. Census Bureau. Each incident was geocoded to latitude and longitude coordinates and spatially joined to the county where it occurred. We summed the number of incidents in each county and then divided by the county population to estimate a county-level rate of fatal police violence per 100,000 residents. This rate was merged with 2020 birth records using the maternal county of residence Federal Information Processing System (FIPS) codes. For the analysis, we modeled fatal police violence both on a categorized scale (in tertiles) and on a continuous scale for sensitivity analyses and monotonic trend tests. Counties with zero recorded incidents of fatal police violence during the exposure window were excluded from the analytic sample, as the analytic focus was on variation among counties with any level of exposure. We acknowledge this limits generalizability and address this in the limitations. The analytic sample was restricted to singleton births to non-Hispanic Black individuals (n = 407,840). Records missing any outcome or key covariate values were excluded.

### Outcomes

Two binary outcomes were examined: first, LBW, defined as birth weight < 2,500 g, and second, PTB, defined as gestational age < 37 weeks. Both were derived from birth certificate records and analyzed separately.

### Covariates

Models adjusted for individual-level maternal covariates based on prior literature and theoretical relevance: maternal age (< 20, 20–24, 25–29, 30–34, 35 +), education level (Basic Education identified as less than High School, High School or GED, Some College, Bachelor’s, Advanced Degree), payment source for delivery (Medicaid, private insurance, self-pay, other), timing of prenatal care initiation (1st–3rd month, 4th–6th month, 7th month to birth, or no care), and WIC participation (yes/no).

### Statistical Analysis

We used Poisson regression models with robust standard errors to estimate incidence rate ratios (IRRs) and 95% confidence intervals for the association between fatal police violence, SREI, and each adverse birth outcome.

We tested interaction effects between fatal police violence and SREI using product terms in fully adjusted models. To account for geographic clustering and unobserved contextual variance, we additionally fit multilevel Poisson mixed-effects models with random intercepts at the county level.

To aid interpretation of interaction effects, we also summarized descriptive county-level characteristics (e.g., housing, education, employment, poverty, incarceration, life expectancy) across groups defined by SREI tertiles and police violence tertiles.

Sensitivity analyses included modeling tertiles of fatal police violence, testing for monotonic trends, and excluding the top 1% and 5% of counties with the highest rates of police violence to assess influence from potential outliers. All analyses were conducted in Stata 19.5.

## Results

### Descriptive Statistics

Among Non-Hispanic Black birthing people in 2020 (n = 407,840), 12.8% experienced preterm birth and 12.8% had low birth weight infants (see Table 1). The mean county-level rate of fatal police violence was 1.78 incidents per 100,000 population, with considerable variation across counties (range: 0.19 to 7.71). Approximately 48% of the sample had basic education, 64% used Medicaid for delivery, and nearly 68% began prenatal care in the first trimester (see Table 1). While the initial sample included 407,840 singleton births to non-Hispanic Black individuals. After excluding records with missing values for key covariates, the final analytic sample for all adjusted models was 403,778.

**Table 1:** Descriptive characteristics of the final analytic sample of black birthing people in the United States (N = 403,778)

Variable	Frequency	Percent
*Adverse Birth Outcomes*		
Low Birth Weight	51,509	12.76
Preterm Birth	51,725	12.81
*County-Level Exposures*		
Police Violence Tertiles		
*Low (T1)*	139,027	34.43
*Medium (T2)*	130,871	32.41
*High (T3)*	133,880	33.16
Structural Disadvantage (SREI) Tertiles		
*Low (T1)*	89,704	22.22
*Medium (T2)*	138,819	34.38
*High (T3)*	175,255	43.40
*Maternal Characteristics*		
Education		
*Basic*	195,106	48.32
*Some College*	131,173	32.49
*Bachelor’s*	50,310	12.46
*Advanced*	27,189	6.73
Maternal Age		
< *20*	25,929	6.42
*20–24*	93,897	23.25
*25–29*	119,603	29.62
*30–34*	97,412	24.13
*35* +	66,937	16.58
When Prenatal Care Began		
* 1 st Trimester*	275,374	68.2
*2nd Trimester*	93,089	23.05
*3rd Trimester*	23,607	5.85
*No care*	11,708	2.9
Delivery Payment Source		
*Medicaid*	259,615	64.3
*Private Insurance*	124,493	30.83
*Self-pay*	8,589	2.13
*Other*	11,081	2.74
WIC Participation		
*No*	211,086	52.28
*Yes*	192,692	47.72

The final analytic sample is conditional upon maternal residence within the 972 U.S. counties with documented exposure to fatal police violence during the 2015–2019 baseline exposure window. SREI percentiles were calculated at the national level prior to sample restriction, leading to variations across tertiles within this conditional context

### Police Violence and Birth Outcomes

Compared to those residing in counties in the lowest tertile (T1) of fatal police violence, Black birthing people living in the highest tertile (T3) experienced a 14% greater risk of low birth weight (IRR: 1.14; 95% CI: 1.12, 1.16) and a 12% greater risk of preterm birth (IRR: 1.12; 95% CI: 1.10, 1.14), with both associations reaching statistical significance (Table 2). In fully adjusted models that did not include interaction terms (Table 2), fatal police violence remained a statistically significant risk factor. Black birthing people living in counties in the highest tertile (T3) of police violence exposure had a 4% greater risk of low birth weight (IRR: 1.04; 95% CI: 1.02–1.06) and a 3% greater risk of preterm birth (IRR: 1.03; 95% CI: 1.01–1.05) compared to those in the lowest tertile (T1).

**Table 2:** Unadjusted and adjusted associations of county-level exposures with adverse birth outcomes (N = 403,778)

Predictor	Low Birth Weight *Unadjusted IRR (95% CI)*	Low Birth Weight *Adjusted IRR (95% CI)*	Preterm Birth *Unadjusted IRR (95% CI)*	Preterm Birth *Adjusted IRR (95% CI)*
*Police Violence Tertiles*				
Low (T1)	1.00 (ref)	1.00 (ref)	1.00 (ref)	1.00 (ref)
Medium (T2)	1.060 (1.039–1.081)***	0.99 (0.97–1.01)	1.058 (1.037–1.079)***	1.00 (0.98–1.02)
High (T3)	1.142 (1.120–1.165)***	1.04 (1.02–1.06)***	1.120 (1.098–1.142)***	1.03 (1.01–1.05)**
*Structural Disadvantage (SREI) Tertiles*				
Low (T1)	1.00 (ref)	1.00 (ref)	1.00 (ref)	1.00 (ref)
Medium (T2)	1.150 (1.123–1.177)***	1.11 (1.08–1.14)***	1.140 (1.114–1.167)***	1.12 (1.09–1.15)***
High (T3)	1.263 (1.235–1.291)***	1.20 (1.17–1.23)***	1.236 (1.209–1.263)***	1.22 (1.19–1.25)***

IRR: Incidence Rate Ratio; CI: Confidence Interval; SREI: Structural Racism Exposure Index. Adjusted Models include covariates maternal age, maternal education, payment source for delivery, prenatal care initiation, and WIC participation

^***^p < 0.001

### Structural Disadvantage and Birth Outcomes

Structural disadvantage was also a significant and consistent predictor of adverse birth outcomes. In unadjusted models, compared to those living in counties in the lowest tertile of structural disadvantage (T1), Black birthing people in the middle tertile (T2) had a 15% higher risk of low birth weight (IRR: 1.15; 95% CI: 1.12–1.18) and a 14% higher risk of preterm birth (IRR: 1.14; 95% CI: 1.11–1.17) (Table 2). Those in the highest tertile (T3) had a 26% higher risk of low birth weight (IRR: 1.26; 95% CI: 1.24–1.29) and a 24% higher risk of preterm birth (IRR: 1.24; 95% CI: 1.21–1.26) (Table 2). These associations remained statistically significant after adjusting for maternal age, education, prenatal care timing, WIC participation, and insurance status. In fully adjusted multilevel models, these strong associations persisted. Individuals in the highest tertile of structural disadvantage had a 25% increased risk of low birth weight (IRR: 1.25; 95% CI: 1.17–1.33) and a 25% increased risk of preterm birth (IRR: 1.25; 95% CI: 1.17–1.33) relative to those in the lowest tertile (Table 2).

### Compounding Risk and Multilevel Findings and Spatial Context

To test whether structural disadvantage modified the link between police violence and birth outcomes, we examined interaction terms within our fully adjusted multilevel models (Table 3).

**Table 3:** Multilevel poisson regression models for the interaction of police violence and structural disadvantage on birth outcomes (N = 403,778)

Variable	Low Birth Weight	Preterm Birth
	*Adjusted IRR (95% CI)*	*Adjusted IRR (95% CI)*
Main Effects		
*Police Violence Tertiles (ref: Low)*		
Medium	0.99 (0.90–1.08)	0.94 (0.85–1.04)
High	1.07 (0.96–1.18)	0.96 (0.87–1.06)
*Structural Disadvantage Tertiles (ref: Low)*		
Medium	1.15 (1.08–1.22)***	1.10 (1.03–1.17)**
High	1.25 (1.17–1.33)***	1.25 (1.17–1.33)***
Interaction Effects (ref: Low PV x Low SREI)		
Medium PV x Medium SREI	1.02 (0.91–1.14)	1.08 (0.96–1.23)
Medium PV x High SREI	0.97 (0.87–1.08)	1.02 (0.90–1.16)
High PV x Medium SREI	0.98 (0.87–1.10)	1.15 (1.01–1.30)*
High PV x High SREI	0.96 (0.86–1.08)	1.06 (0.95–1.19)
County-Level Variance	0.009***	0.016***

IRR: Incidence Rate Ratio; CI: Confidence Interval; PV: Police Violence; SREI: Structural Racism Exposure Index ^a^ All models are adjusted for maternal age, education, WIC participation, prenatal care initiation, and payment source for delivery. ^b^ The county-level variance represents the random intercept variance, which was statistically significant (p < 0.001) in all models, justifying the use of a multilevel approach

^*^p < 0.05, **p < 0.01, ***p < 0.001

For low birth weight, we found no evidence of a combined association. None of the interaction terms between police violence tertiles and structural disadvantage were statistically significant. For instance, the IRR for residing in a county with high police violence and high structural disadvantage was 0.96 (95% CI: 0.86–1.08) compared to the reference group of low police violence and low structural disadvantage.

In models predicting preterm birth, however, the results support the hypothesis of a combined association. We found a statistically significant interaction between high police violence and medium structural disadvantage. Black birthing people in these counties had a 15% greater risk of preterm birth compared to those in the reference group (IRR: 1.15; 95% CI: 1.01–1.30). This finding indicates that the risk of preterm birth is significantly elevated in counties characterized by the dual exposures of high fatal police violence and medium levels of structural disadvantage (Table 3).

Descriptive contrasts revealed that counties in the medium SREI/high PV group had moderate structural disadvantage (average SREI ≈ 0.22, life expectancy ≈ 77 years) and elevated fatal police violence rates (≈ 2.7 per 100,000), compared to high SREI/high PV counties (SREI ≈ 0.95, life expectancy ≈ 75 years, PV ≈ 3.1) and low SREI/low PV counties (SREI ≈ –0.71, life expectancy ≈ 80 years, PV ≈ 0.7).

To account for geographic clustering and provide the most robust test of our hypothesis, we examined the interaction between police violence and structural disadvantage using multilevel Poisson models with random intercepts for counties (n = 972). Multilevel models yielded similar results: no significant interactions for LBW, and a single significant interaction for PTB (high police violence × medium structural disadvantage).

### Sensitivity and Robustness Checks

A series of supplementary analyses using alternative model specifications and coding strategies was conducted to assess the robustness of our findings. First, we re-estimated our models using a continuous specification of county-level police violence rather than categorical tertiles. Poisson regression models included main effects and interaction terms with structural disadvantage. Results were substantively consistent with the primary analyses: while continuous PVrate was not significantly associated with LBW or PTB on its own, the interaction between PVrate and high SREI was statistically significant for PTB (IRR:1.0341, 95% CI: 1.0058–1.0625), indicating that police violence on PTB risk may be heightened in contexts of high structural racism (Table 4).

**Table 4:** Sensitivity analyses using a continuous measure of police violence and excluding outlier counties

Model Specification	Low Birth Weight	Preterm Birth
	Adjusted IRR (95% CI)	Adjusted IRR (95% CI)
Police Violence as Continuous Variable		
Per 1-unit increase in rate	1.022 (1.013–1.031)***	1.024 (1.016–1.032)***
Excluding Top 1% of Counties by Police Violence Rate		
Per 1-unit increase in rate	1.026 (1.017–1.035)***	1.028 (1.019–1.037)***
Excluding Top 5% of Counties by Police Violence Rate		
Per 1-unit increase in rate	1.018 (1.007–1.029)***	1.015 (1.004–1.026)**

IRR: Incidence Rate Ratio; CI: Confidence Interval. < sup > a </sup > All models are adjusted for maternal age, maternal education, structural disadvantage (SREI), payment source for delivery, prenatal care initiation, and WIC participation

^**^p < 0.01, ***p < 0.001

Additionally, we fit multilevel Poisson models with random intercepts for state-county clusters to account for geographic variation in unmeasured contextual factors. These models showed robust associations between high structural disadvantage and adverse birth outcomes. At the same time, the interaction between police violence and SREI persisted only for PTB (IRR: 1.1451, 95% CI: 1.0067–1.3025 for high PVrate and medium SREI). Model comparison using AIC and BIC confirmed a better fit in the multilevel specification.

Together, these sensitivity checks support the robustness and stability of our primary findings and affirm that the intersection of structural racism and state violence is consequential for Black perinatal health.

## Discussion

This study affirms that for Black birthing people in the United States, county-level exposure to fatal police violence, measured categorically in tertiles, is associated with a statistically significant but modest increase in relative risk of both LBW and PTB. Structural disadvantage showed strong and consistent associations with these adverse birth outcomes. Even after accounting for key individual-level covariates such as maternal age, education, prenatal care timing, and insurance status, both police violence and structural disadvantage were associated with adverse birth outcomes in adjusted models. However, the association between police violence and birth outcomes attenuated and became non-significant in multilevel models that accounted for county-level clustering, indicating spatial and contextual variation in these relationships, particularly in urban and urban-adjacent counties where policing intensity and structurally produced disadvantage are often concentrated.

These findings are consistent with what public health scholars, community organizers, and reproductive justice advocates have long advocated for contextualizing policing as a public health issue, and acknowledging the harms it inflicts extend beyond physical violence to the stress, surveillance, and institutional neglect that shape Black life [2, 30–32]. For Black birthing people, encounters with state violence are not limited to moments of crisis; they are embedded in the everyday conditions of life, especially in urban communities where police presence, surveillance, and structurally produced stressors are densely patterned. These chronic exposures may create a physiological environment of stress and instability that undermines reproductive health long before conception [1, 2, 8, 24].

This study also underscores the importance of understanding structural racism and police violence as intersecting, not merely additive, exposures. Across all models, including adjusted and multilevel specifications, structural disadvantage is consistently and robustly associated with low birth weight and preterm birth. Interaction models revealed that the combination of high structural disadvantage and elevated police violence was associated with a statistically significant increase in the risk of preterm birth in one overlapping exposure context (high PVrate × medium SREI), with other interaction terms approaching but not reaching statistical significance. These patterns offer suggestive, but not conclusive, evidence of potential compounded structural risk, particularly in urban settings where structural disadvantage and policing often intersect within the same spatial and institutional landscape (Table 3). This finding supports the hypothesis that compounded structural harms, those that simultaneously operate through community-level racism and carceral violence, may intensify reproductive risk. Again, these findings should be interpreted as exploratory and suggestive rather than definitive evidence, warranting further investigation in future research.

To further interpret these interaction findings, descriptive contrasts across county groups offer additional insight. Counties in the medium SREI/high PV group displayed moderate levels of structural disadvantage, with some stability in domains like housing, education, and wealth, yet elevated rates of fatal police violence. These conditions may make the disruptive association of lethal police violence particularly acute. By contrast, in the most disadvantaged counties (high SREI/high PV), reproductive risks may already be so saturated by entrenched poverty, incarceration, and housing instability that additional exposure to police violence does not measurably increase outcomes. In low SREI/low PV counties, resources and protections may buffer communities from these harms. This interpretation underscores that police violence and structural disadvantage, while correlated, are not interchangeable exposures; their relationship is context-dependent and shaped by the social ecology of place, including the urban policy environments and concentrated structural conditions that shape health opportunities and stressors in many Black communities.

Importantly, this work resists the dominant epidemiologic norm of using White populations as a benchmark for health [33]. Instead, we center Black birthing people as the analytic focus, shifting the lens toward understanding how structurally produced harm operates within Black communities. This approach provides a clearer view of within-group variation and reflects broader calls to examine racism directly as a central exposure [34]. Recent scholarship emphasizes that counterfactual models using race as a stand-in for racism risk obscuring White privilege and misrepresenting how racism operates in practice [35].

This study is also informed by the principles of Reproductive Justice and Black Feminist Thought, which recognize that the ability to have children, not have children, and raise children in safe and sustainable environments is inseparable from the structural conditions that shape Black life [26, 36, 37]. These frameworks challenge dominant public health paradigms that use Whiteness as the normative reference group and instead call for analytic approaches that center the lived experiences of Black people. Our decision to conduct an intra-racial analysis, focusing exclusively on non-Hispanic Black birthing people, reflects this orientation. Rather than masking within-group variation through cross-racial comparisons, intra-racial analysis allows us to examine how place, policy, and exposure to state violence shape reproductive harm within Black communities themselves [33].

This analytic choice is not only methodological, but also political and epistemological. Black Feminist epistemology teaches us that those most impacted by systems of harm are best positioned to understand them [36]. This aligns with Reproductive Justice’s call to investigate how reproductive outcomes are shaped not simply by access to care, but by structural systems such as policing, housing, and economic deprivation [26]. By examining variation within Black populations across counties, this study underscores that reproductive outcomes are fundamentally place-based, policy-shaped, and justice-linked.

The county-level variation further affirms the need to consider place and spatial context in future research. Consistent with earlier models, structural disadvantage remained a robust predictor. In contrast, associations between police violence and birth outcomes attenuated in multilevel models, highlighting the influence of spatial clustering and contextual variation. These results point to the value of integrating geospatial analysis. Future work may explore spatial heterogeneity in greater depth by incorporating neighborhood-level data or examining local policy variations that may moderate these associations.

### Limitations

This study is not without limitations. First, the MPV database, while widely regarded as the most comprehensive source of fatal police violence incidents in the U.S., captures only deaths resulting from police encounters. As such, it does not account for the full continuum of state violence, including non-lethal but harmful exposures like police stops, searches, harassment, threats, or surveillance, each of which may generate chronic stress that negatively affects reproductive health [2, 4, 8]. By capturing only fatal outcomes, MPV likely underestimates the true scope and burden of police violence, particularly the cumulative associations of repeated or indirect exposures within communities.

Additionally, MPV reflects police encounter outcomes, not processes or contexts. It lacks individual exposure timing data and omits unreported police killings, risking misclassification bias, especially in resource-limited counties. Next, our main exposure, county-level police fatalities, was highly skewed, with few counties responsible for most incidents. Since only counties with at least one fatal encounter were included, true zero-inflation wasn't observed, but the rarity and clustering raised concerns. We performed tertile analyses, trend tests, and sensitivity checks, excluding top PV rate counties. Future research could use rate-smoothing or hierarchical spatial models.

Initially, SREI was at the census tract level; we aggregated it to counties, which may mask local disparities. Finally, the 2020 birth cohort coincided with COVID-19 and racial protests, potentially influencing outcomes and perceptions. Despite the cross-sectional design limiting causal claims, consistent findings support validity. Future work should explore longitudinal data, non-fatal encounters, and finer geographic units for more nuanced insights.

Ultimately, this paper adds to the growing body of evidence that connects structural racism and disadvantage and police and state violence to perinatal health [20, 23, 23, 24, 38]. But it also pushes beyond documenting disparity; it underscores the urgency of addressing and ultimately dismantling systems of racialized harm and investing in models of care, safety, and accountability rooted in reproductive justice. Addressing inequities in birth outcomes requires more than improved clinical care. It demands a reckoning with how structural conditions and the institutions that maintain them produce and reproduce harm. This reckoning must include reimagining a world in which we divest from systems of surveillance and punishment and reinvest those resources in housing, education, transportation, doula support, and other social and structural determinants of health. It calls for critical attention to the over-policing of Black communities, and for reparative policies that acknowledge and atone for the historical and ongoing harms enacted against Black birthing people and their families. A just future for reproductive health demands nothing less than a transformative commitment to healing, accountability, and systemic change.

## Data Availability

The individual-level birth certificate data analyzed in this study were obtained from the National Center for Health Statistics (NCHS) restricted-use natality files. Because of strict federal confidentiality restrictions, these data are not publicly available and cannot be shared by the authors. Researchers interested in accessing these data must apply directly to the National Center for Health Statistics (NCHS). Police Violence project database (https://mappingpoliceviolence.org/). The Structural Racism Exposure Index (SREI) census-tract data were originally developed by Dyer et al. (23) and can be requested from the original authors or reconstructed using public U.S. Census Bureau and American Community Survey files.
